# Evaluation of an alternative centrifugation protocol for reducing total turnaround time

**DOI:** 10.1515/almed-2024-0170

**Published:** 2024-11-21

**Authors:** Reyes Nicolás de Blas, Miriam Menacho Román, Sara Olivares Romero, Claudia Mesas Mariñán, Alba Arroyo Vega, Graciela Martín Gómez, María Álvarez Pastor, Lucía Castillo Menéndez, María José Azofra Villa, María del Pilar Pérez Sobrino, Ignacio Arribas Gómez

**Affiliations:** Servicio de Bioquímica Clínica, Hospital Universitario Ramón y Cajal, Madrid, Spain; Extracciones Centrales, Hospital Universitario Ramón y Cajal, Madrid, Spain

**Keywords:** centrifugation, turnaround time, preanalytical

## Abstract

**Objectives:**

Centrifugation is a key process that should be controlled to ensure an adequate sample quality. To achieve rapid, standardized, and consistent sample centrifugation, we aimed to evaluate an alternative centrifugation protocol and its impact on the results of 20 biochemical determinations in serum.

**Methods:**

The study included 45 ambulatory patients. Two serum-separating tubes were collected from each patient (Becton Dickinson (BD) Vacutainer^®^ SST™ II Advance, 8.5 mL Ref. 366468)*.* One of the tubes was centrifuged at 2,530 ×  *g* for 10 min (control method), while the other tube was centrifuged under alternative centrifugation conditions, namely 2,530 ×  *g* for 7 min.

**Results:**

The analysis of results revealed that calcium, total bilirubin, and magnesium exhibited a constant and proportional systematic bias. However, considering the proportional mean difference, all analytes met the desirable bias performance established by our laboratory, except for magnesium, which met the minimum bias criteria.

**Conclusions:**

Our study demonstrates that both centrifugation protocols are interchangeable for measuring the studied analytes, thereby ensuring adequate sample quality.

## Introduction

Centrifugation is a pre-analytical procedure repeatedly performed in routine clinical laboratory practice. However, there is paucity of data documenting the influence of centrifugation on laboratory results [1], [2], [3], [4], [5], [6]. Centrifugation is a key process that should be controlled to ensure adequate sample quality.

Laboratories are required to establish a standardized centrifugation protocol. This protocol should define the optimal relative centrifugal force (RCF), temperature, and time required to obtain high-quality samples for each type of tube, based on the manufacturer’s recommendations and turnaround time (TAT) requirements. Any deviation from these recommendations requires prior validation [7].

An adequate TAT is crucial for clinical laboratory quality. The laboratory is expected to provide rapid, reliable, and accurate reports enabling appropriate and timely medical decision-making. TAT is considered as a key performance indicator in quality assurance programs. In fact, routine TAT measurement and evaluation are essential to ensure extra-analytical quality. TATs are recorded and reviewed to adopt corrective measures, should it be necessary [8].

General centrifugation recommendations for the serum biochemistry parameters analyzed are 1000–3000 ×  *g* for 10–15 min at a temperature of 15 to 24 °C [7]. There are validated alternative centrifugation procedures involving a higher RCF and a shorter centrifugation time, resulting in shorter TATs [7].

The alternative centrifugation conditions (3,000 ×  *g*-5 min) validated by Becton Dickinson (BD) in the *BD White Paper* VS*7228 study* [9], were proven to be interchangeable with control conditions (1,300 ×  *g*-10 min). In our case, since 3000 ×  *g* centrifugation was not recommended by the manufacturer (Kubota S700TR), centrifugation was performed at 2,530 ×  *g* (maximum speed available) for 7 min [9].

The purpose of this study was to establish a harmonized, standardized, and faster centrifugation protocol for all sample types without significantly affecting plasma/serum quality. As a first approach, the study was conducted only with serum samples for biochemical parameter determination.

In this study, we aimed to assess the clinical performance of BD Vacutainer^®^ SST™ II Ad Advance tubes under centrifugation conditionsof 2,530 ×  *g* for 7 min. Control centrifugation conditions were set at 2,530 ×  *g* for 10 min.

## Materials and methods

The study was conducted by the Department of Clinical Biochemistry of Ramón y Cajal University Hospital (HURYC) between May and June 2024.

The project was approved by the HURYC Ethics Committee under code 046/24.

The sample consisted of 45 ambulatory male and female patients with ages ranging from 18 to 70 years. Informed consent was obtained from all participants. Cancer patients were excluded.

For each patient, two serum-separating tubes were collected (Becton Dickinson (BD) Vacutainer^®^ SST™ II Advance, 8.5 mL Ref. 366468). Blood collection was performed using a Vacuette safety-lok 21G set (0.80 ×19 mm) with a pre-attached holder (Ref. 450085V1 Greiner Bio-One*)* following the Clinical and Laboratory Standards Institute (CLSI) GP41 guidelines [10] and the joint guidelines of the European Federation of Clinical Chemistry and Laboratory Medicine (EFLM) and the Latin America Confederation of Clinical Biochemistry [11].

All sample pairs were handled under the same pre-analytical conditions (collection, shipping, centrifugation time and temperature) to limit interference from other variables.

Samples were stored at room temperature in vertical position for a minimum of 30 min to ensure complete clot retraction.

All samples were centrifuged in a Kubota S700TR swinging-bucket rotor centrifuge (Rotor RS-1440 M) (preventive maintenance was performed prior to initiation of the study). As a control method, one of the two samples from each patient was centrifuged at 2,530 ×  *g* for 10 min. The other tube was centrifuged at alternative centrifugation conditions, namely 2,530 ×  *g* for 7 min. All samples were centrifuged at room temperature (18–22 °C). The order of tube collection and centrifugation protocol for each group was randomized.

A total of 20 biochemical parameters (Table 1) were analyzed on an Alinity c analyzer (Abbott Diagnostics, IL, USA). Samples were processed in duplicate immediately after centrifugation.

**Table 1:** Comparison of the results obtained with the two centrifugation conditions. Descriptive study.

Analyte	Units	n	Study range	Mean ± SD/median [p25-p75]	
				2530 × *g* 10 min	2530 × *g* 7 min
Glucose	mg/dL	44	72–142	95.66 ± 14.27	95.90 ± 14.47
Sodium	mmol/L	45	136–145	140.59 ± 2.02	140.62 ± 2.15
Potassium	mmol/L	45	3.71–6.57	4.54 ± 0.47	4.51 ± 0.45
Chlorine	mmol/L	45	100–114	106.44 ± 2.89	106.31 ± 2.97
Creatinine	mg/dL	45	0.53–2.82	1.02 ± 0.45	1.02 ± 0.45
Phosphate	mg/dL	44	2.29–4.63	3.37 ± 0.59	3.36 ± 0.59
Calcium	mg/dL	45	8.5–10.9	9.60 ± 0.48	9.64 ± 0.50
Cholesterol	mg/dL	45	107–256	178.74 ± 37.74	178.49 ± 37.41
Total protein	g/dL	45	6.25–8.05	7.08 ± 0.37	7.08 ± 0.36
Total bilirubin	mg/dL	45	0.24–2.69	0.66 ± 0.40	0.66 ± 0.40
Alkaline phosphatase	U/L	45	36–173	81.59 ± 72.78	81.28 ± 27.74
Alanine aminotransferase	U/L	44	7–50	20.56 ± 9.67	20.55 ± 9.65
Lactate dehydrogenase	U/L	45	92–317	187.94 ± 37.88	188.21 ± 39.29
Iron	µg/dL	45	28–159	88.30 ± 31.79	88.31 ± 31.86
Magnesium	mg/dL	45	1.53–2.34	1.94 ± 0.16	1.97 ± 0.15
Urea	mg/dL	44	18–126	33.25 [28.50 to 45.75]	33.00 [28.50 to 46.25]
Gamma glutamyltransferase	U/L	45	11–247	25.00 [20.37 to 36.62]	25.5 [20.00 to 36.00]
Aspartate aminotransferase	U/L	45	11–51	20.00 [17.87 to 23.12]	21.00 [17.87 to 23.50]

SD, standard deviation; p25, 25th percentile; p75, 75th percentile.

### Statistical analysis

Outliers were excluded according to the recommendations in the CLSI EP09-A2 guidelines [12].

Descriptive analysis of quantitative variables was performed using mean and standard deviation for parametric variables and median with 25th and 75th percentiles for non-parametric variables.

The Kolmogorov-Smirnov test was used to assess the normal distribution of data.

Once the linear relation between the series was confirmed, the level of agreement between the two protocols was evaluated using the Bland-Altman [13] plot and Lin’s concordance correlation coefficient [14].

In accordance with our analytical performance specifications, the maximum allowable systematic error (SE) was based on desirable biological variation (BV) (between- and within-subject variation, CVI and CVG), obtained from the EFLM database, using the following formula [15]:

$$Bias<0.25\times {\left({CVI}^{2}+{CVG}^{2}\right)}^{1/2}$$

Estimations were performed using MedCalc^®^ 12.3 (MedCalc Software, Ostende, Belgium). A p-value<0.05 was considered statistically significant.

## Results

Uric acid and triglyceride determinations were excluded, as they exceeded the allowable number of outliers for comparison. For all other parameters, adequate values were obtained in a minimum of 44 subjects (Table 1).

Bland-Altman plots revealed that calcium, total bilirubin, and magnesium showed a constant and proportional systematic bias, as the 0 value was not included in any of the confidence intervals generated for absolute and relative differences. However, considering the proportional mean difference, all analytes met the desirable bias requirement accepted by our laboratory, except for magnesium.

**Figure 1:**
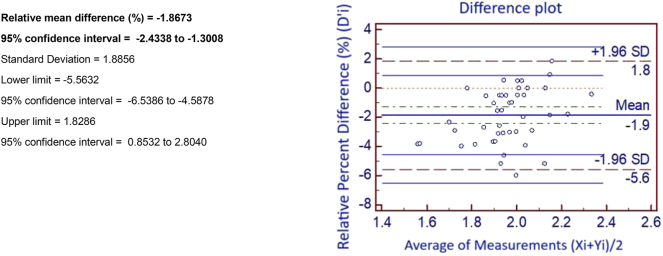
Results of Bland-Altman test for magnesium, provided as an example. Yi: mean value for magnesium obtained with the evaluated method (7 min). Xi: mean value for magnesium obtained with the control method (10 min). D’i: D’i percent relative difference: Xi-Yi/[(Xi+Yi)/2]*100. Data are commented in the manuscript.

As shown in Figure 1, the alternative centrifugation method consistently produced slightly higher magnesium values compared to the control method. This difference kept constant in all the concentrations studied. However, considering biological criteria and despite not meeting the desirable bias criterion, the relative differences obtained for magnesium were lower than the minimum bias recommended by the EFLM [15].

The results obtained for all the analytes included in the study are displayed in Tables 1 and 2.

**Table 2:** Comparison of the results obtained with the two centrifugation conditions. Agreement analysis.

Analyte	Bland-Altman	Limit of maximum acceptable difference, %	Lin’s concordance correlation coefficient
	Relative mean difference (95 %CI)		
Glucose	0.26 (−0.18 to 0.70)	1.2	0.9985
Sodium	−0.03 (−0.17 to 0.13)	0.1	0.9421
Potassium	0.43 (−0.64 to 1.51)	0.8	0.9358
Chlorine	−0.13 (−0.26 to 0.01)	0.2	0.9855
Creatinine:	0.45 (−0.12 to 1.01)	2.1	0.9991
Phosphorus	0.22 (−0.23 to 0.66)	1.6	0.9970
Calcium	**−0.42 (−0.65 to −0.19)**	**0.8**	0.9847
Cholesterol	0.11 (−0.20 to 0.43)	2	0.9987
Total protein	−0.02 (−0.35 to 0.30)	0.5	0.9774
Total bilirubin	**−0.71 (−1.36 to −0.06)**	**4**	0.9995
Alkaline phosphatase	0.40 (−0.01 to 0.81)	2.7	0.9991
Alanine aminotransferase	0.23 (−0.74 to 1.20)	5	0.9984
Lactate dehydrogenase	−0.07 (−1.55 to 1.41)	1.6	0.9626
Iron	−0.02 (−0.59 to 0.56)	4.8	0.9985
Magnesium	**−1.87 (−2.43 to −1.30)**	**2.4**	0.9448
Urea	0.14 (−0.31 to 0.59)	3.1	0.9997
Gamma glutamyltransferase	0.71 (−0.09 to 1.51)	5.7	0.9998
Aspartate aminotransferase	−0.98 (−2.18 to 0.22)	2.7	0.9941

The desirable bias (%) was established as the allowable limit, except for magnesium (minimum bias)- EFLM database. Lin’s concordance correlation coefficient>0.99: almost perfect agreement; 0.95–0.99: very strong agreement; 0.90–0.95. Moderate agreement; <0.90: poor agreement. 95  %CI, 95  % confidence interval. Results for calcium, total bilirubin and magnesium, as discussed in the text, are indicated in bold.

## Discussion

To ensure an adequate TAT, centrifugation conditions can be adjusted as long as sample quality is not compromised.

Following BD-validated recommendations [9], we proposed and evaluated an alternative centrifugation protocol that was feasible in our laboratory (2,530 ×  *g* for 7 min).

Multiple studies have demonstrated that alternative centrifugation times and RCFs using different collection tubes and instruments can provide equivalent analytical precision [1], [2], [3], [4], [5]. There is cumulative evidence that centrifugation time can be reduced below 10 min without negatively impacting sample quality for most parameters studied. However, differences in the recommended centrifugation conditions limits the generalization of results from studies using a specific collection tube to tubes from other manufacturers. Other studies demonstrate sample quality variations with RCF exceeding 4000 ×  *g* [6].

Further studies are needed to assess the role of other factors related to sample quality, such as gel barrier formation and serum separation as examined by BD in the study *BD White Paper* VS*7228* [9]. Other factors to consider include the presence of residual fibrin or cells in serum/plasma.

Laboratories using centrifugation conditions different from those recommended by the tube and/or reagent manufacturer should first perform a validation study.

In our laboratory, near 3000 samples are centrifuged daily in approximately 40 centrifugation runs, of which 28 % are preferential. Theoretically, a 3-min reduction of centrifugation time would save 120 min per day. A follow-up study is needed to confirm this TAT reduction in real practice.

Given the high sample volume requiring centrifugation and the need for rapid results, the 3-min reduction in the centrifugation protocol is justified.

The results of this study confirm that the two centrifugation protocols are interchangeable for measuring the analytes studied, thereby ensuring adequate sample quality.

A larger-scale study including other parameters and specimens (plasma) would be needed to obtain more robust conclusions.
